# Membranous Nephropathy: From Research Bench to Personalized Care

**DOI:** 10.3390/jcm10061205

**Published:** 2021-03-14

**Authors:** Barbara Moszczuk, Krzysztof Kiryluk, Leszek Pączek, Krzysztof Mucha

**Affiliations:** 1 Department of Immunology, Transplantology and Internal Diseases, Medical University of Warsaw, 02-006 Warsaw, Poland; barbara.moszczuk@wum.edu.pl (B.M.); leszek.paczek@wum.edu.pl (L.P.); 2 Department of Clinical Immunology, Medical University of Warsaw, 02-006 Warsaw, Poland; 3 Department of Medicine, Division of Nephrology, Vagelos College of Physicians and Surgeons, Columbia University, New York, NY 10032, USA; kk473@cumc.columbia.edu; 4 Institute of Biochemistry and Biophysics, Polish Academy of Sciences, 02-106 Warsaw, Poland

**Keywords:** membranous nephropathy, glomerulonephritis, personalized medicine, PLA_2_R, proteomics

## Abstract

Membranous nephropathy is a glomerulopathy that causes nephrotic syndrome and, in at least a third of cases, lasting end-stage kidney disease (ESKD). It is also a rare case of revolutionary changes in our understanding of the disease, that translates from scientific findings to real diagnosis and treatment recommendations in less than ten years. In this review we present: (1) a short history and traditional approach to patients with membranous nephropathy, (2) current recommendations and treatment options that have emerged in recent years, (3) findings of new studies, with a particular focus on serological/immunological methods, genomic and proteomic studies, still requiring validation. With further development in this field, membranous nephropathy may become one of the first nephrological conditions that apply a truly personalized approach with the omission of invasive measures such as kidney biopsy.

## 1. Introduction

Membranous nephropathy (MN) is a rare cause of nephrotic syndrome. The disease starts with the accumulation of immune deposits in the subepithelial space with local complement activation and damage to podocytes and the glomerular basement membrane. Between 70% and 80% of MNs have been classified as idiopathic or primary (pMN), with the remainder is related to secondary causes (sMN), such as malignancies, infections or autoimmune diseases. This classification is important, because these two types trigger different management approaches; however, it is likely to be altered as specific antigens for each disease subtype are being discovered. In primary MN, spontaneous remission occurs in approximately 30% of patients, while the remaining patients have persistent proteinuria or progression to the end stage kidney disease (ESKD). The most severe cases of pMN are treated with immunosuppression (IS). Secondary MN generally requires treatment of the underlying diseases. In this article, we review the traditional approach to MN and summarize recent improvements in diagnostics, risk stratification, and the treatment of MN and its co-morbidities.

## 2. The Hunt for MN Autoantigens—A Brief History

The search for an antigen that triggers an immunological reaction dates back to the late 1950s, when Walter Heymann conducted an experiment in which he administered a concoction of rat kidney cortexes, containing antigens from the brush border of proximal tubules, to mice. Rodents given this mixture presented severe nephrotic syndrome. In the 1970s and 1980s, scientists developed a so-called “passive” model. Immunoglobulin G (IgG) from rats with Heymann nephritis was injected into healthy rats, resulting in the development of heavy proteinuria. Immunoglobulins came from animals immunized with megalin. In this model, transferred antibodies bind to an intrinsic megalin (autoantigen) on the surface of rat podocytes, inducing complement activation and podocyte injury. However, becuase megalin is not present on human podocytes and thus the identity of the human MN antigen remained unsolved [1].

In 1982, Border et al. hypothesized that negatively charged capillary walls attract positively charged proteins. They used cationic bovine serum albumin (BSA) to immunize rabbits to develop proteinuria, subepithelial deposits of IgG, and complement component 3 [2]. In 2011, Debiec et al. found significantly enhanced levels of anti-BSA antibodies in children below 5 years old. In the immunofluorescent staining of kidney biopsies, BSA and anti-BSA IgG were present in the glomeruli. In this model, an exogenous antigen of dietary origin (BSA) passes the intestinal border in an unchanged form. A small fraction of positively charged proteins is able to bind to the glomerular basement membrane (GBM) and is targeted by circulating antibodies [3].

A particular case of alloimmunity was described in children with perinatal MN. Four infants born with a nephrotic syndrome were found to have antibodies against neutral endopeptidase (NEP) expressed by human podocytes [4]. It was discovered that their mothers lacked a gene necessary for NEP synthesis and, thus, treated the NEP present in their children as the target of an antibody attack. Anti-NEP IgG moved through the placenta and caused MN-like glomerular damage in the newborns. This case demonstrated for the first time that anti-podocyte antibodies caused MN in humans; however, this specific mutation is rare and does not explain adult MN [5]. The historical antigens are summarized in Table 1.

**Figure 1:**
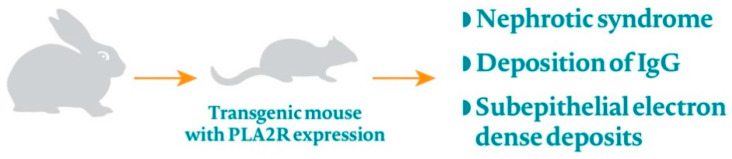
A study by Meyer-Schweinger and Tomas proved that antibodies against phospholipase A2 associated receptor 1 (PLA2R) cause primary membranous nephropathy. Immunoglobulin G (IgG).

The discovery of antibodies against M-type phospholipase A2 receptor (PLA2R) situated on the cell surface of human podocytes has been a turning point for the MN field [6]. Until recently, we lacked experimental evidence that PLA2R was indeed the culprit antigen that triggered the disease, mainly because it is not expressed on mouse podocytes. However, an important study [7] showed that the transfer of anti-PLA2R antibodies to transgenic mice equipped with this receptor caused clinical and pathomorphological results consistent with MN (Figure 1). 

PLA2R is not the only antigen that induces kidney damage in membranous nephropathy. Described in 2014, thrombospondin type 1 domain-containing 7A (THSD7A), was additionally described as another podocyte-expressed auto-target explaining a smaller number of cases [8]. Research estimated that antibodies against this podocyte protein account for ca. 3% of primary MN [9]. The experimental proof for the pathologic role of these antibodies was also generated by the same team [10].

Interestingly, THSD7A is also found in malignant tissues. As many as 28% of patients with THSD7A-assosciated MN were diagnosed with sMN due to an occult malignancy [11]. De Vriese et al. proposed that PLA2R-negative patients with sMN on a kidney biopsy should be screened for THSD7A and, if the test is positive, be subjected to a through oncological evaluation [12]. In cancer patients treated with chemotherapy, THSD7A appeared to disappear at the end of treatment simultaneously with the proteinuria [11]. The confirmed antigens discovered to date are summarized in Table 2. 

## 3. The Traditional Approach to Diagnosis and Treatment

**Figure 2:**
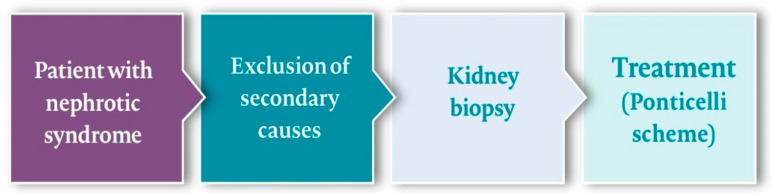
Traditional approach to a patient with MN-related proteinuria.

A patient presenting with nephrotic syndrome, after a screening for secondary causes, typically undergoes a kidney biopsy, which is considered to be the gold standard diagnostic tool (Figure 2).

The name “membranous” describes the characteristic microscopic picture of thickened capillary walls, which results from the subepithelial accumulation of IgG. The deposits give the basement membrane a “spiked” appearance on light microscopy and forms granular lines in immunofluorescent imaging. Electron microscopy is used to confirm electron-dense subepithelial deposits, usually accompanied by the effacement of podocyte foot processes. Once the diagnosis is established, Kidney Disease Improving Gloal Outcomes (KDIGO) advises a 6-month observation period, as there are many cases of spontaneous remission (SR) of MN. However, it typically takes more than a year to achieve SR [13].

This time of watchful waiting can be stressful, as the patient often faces potential complications of nephrotic syndrome including edema, hypoalbuminemia, secondary immunodeficiency associated with the loss of immunoglobulins, and potentially thrombosis. Therefore, most clinicians decide to treat immediately. The standard approach (Ponticelli scheme) consists of a 6-month course of alternating cycles of corticosteroids and the alkylating agents chlorambucil or cyclophosphamide (the latter has a better safety profile) [14]. After the administration of Ponticelli regimen, the patients are followed every 3 to 6 months. Proteinuria is quantified at regular intervals by the urine protein to creatinine ratio or 24-h urinary protein excretion to assess the effectiveness of the treatment. This therapy line has the best evaluation in randomized controlled trials and significantly reduces all-cause mortality and ESKD. However, it is not given lightly due to serious adverse effects, which include bone marrow suppression, infertility, and malignancies. Because of a long-term unfavorable safety profile, there have been several attempts at cyclophosphamide (CYF) replacement. In 2001, the effectiveness of cyclosporin in tackling steroid-resistant MN was reported [4]. Both cyclosporin and tacrolimus seemed to have better short-term efficacy and safety than CYF, but their clinical use is limited as they result in more relapses [15,16].

## 4. Newer Diagnosis and Treatment Algorithms

The detection of serum PLA2R is practically tantamount with the diagnosis of MN, as the specificity of this test at standard cut-off levels of >20 units/mL exceeds 97% [17]. The sensitivity, however, is lower and ranges from 60%–70%. This can be improved by PLA2R and THSD7A staining of biopsy specimens. In a “kidney as a sink” hypothesis, researchers suggested that PLA2R antibodies can sometimes appear in the blood after the kidneys’ buffering capacity is exceeded [18]. This would explain why some patients become seropositive over a longer period of time. Van de Logt et al. proposed a re-examination of PLA2R-Ab after 6 months of follow up [18,19].

Changes in the PLA2R antibodies levels also provide a prognostic value and are helpful in monitoring the disease activity; high titres correlate with proteinuria [20,21], spontaneous remission [22,23], and poor renal outcome [24]. 

Dai et al. demonstrated that some sMN patients may also be PLA2R positive, including cases in the setting of lupus, hepatitis B, or malignancies [25]. In fact, between 3% to 9% of PLA2R positive patients have a concurrent malignancy [26,27]. The occurrence of detectable anti-PLA2R-Ab in serum or biopsy specimens of patients with “secondary” MN is a rare event, and the significance is yet to be established [28]. In the meantime, all MN patients should be screened for secondary causes. An anti-THSD7A test is also now routinely available and should be used alongside PLA2R-Ab to classify the disease by the type of the primary antigen.

After the diagnosis is established, the important clinical question is that of predicting a spontaneous remission during the “watchful waiting” period. As the antibodies have an advantage of preceding the changes in proteinuria, De Vriese et al. proposed an algorithm that helps to undertake clinical decisions by repeated quantification of the PLA2R antibody levels. The suggested thresholds were: 20–86 units/mL (low level PLA2R-Ab), 87–201 units/mL (medium), and ≥202 units/mL (high) [22]. 

**Figure 3:**
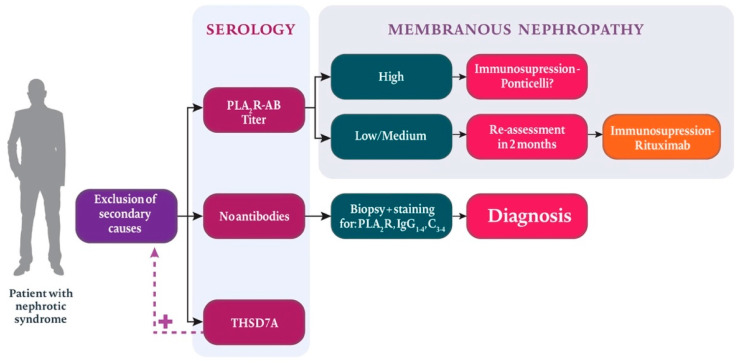
A proposed decision-making algorithm for a patient with nephrotic syndrome. Based on de Vriese and Bomback [29,30].

Patients with high PLA2R-Ab titres and any level of proteinuria are to be re-tested in one month. An increase or persistent high level of antibodies and persistent nephrotic range proteinuria could be an indication to begin IS, provided that the renal damage is not beyond repair. This algorithm appears reasonable, as high levels of PLA2R are associated with a more rapid loss of renal function. However, this algorithm still requires validation in prospective studies (Figure 3). 

Current evidence supports the recommendation to perform PLA2R measurements repeatedly, including at the end of the administered therapy, as the antibody status can be used to predict the treatment effectiveness [27]. The PLA2R levels should also be measured in ESKD patients who await kidney transplantation, as the levels appear to correlate with MN recurrence after transplantation [31]. 

Antibodies to target antigens PLA2R and THSD7A explain approximately 60%–70% of primary MN cases. In recent years, a number of other additional potential antigens have been identified. Antibodies against neural epidermal growth factor-like 1 protein (NELL-1) were present in biopsy tissue in 3.8% [32] or even 23% of PLA2R/THSD7A negative patients [33]. Another antigen, protocadherin, was found in 9% of non-PLA2R/THSD7A biopsies [34]. Both antigens appear to be linked to MN occurring in older patients. In the paediatric and young adult population, antibodies against semaphorin 3B were described in 11 cases of MN [35]. The discovery of new target antigens in MN is likely to make us revisit the definitions of primary and secondary MN. Sometimes, however, membranous changes in the kidneys and subsequent proteinuria can appear well before any other signs of autoimmunity come into light. For example, exostosin-1 and 2 have been described in patients with a subtype of MN associated with autoimmune diseases, particularly lupus nephritis [36].

Other novel autoantigens found in patients with lupus nephritis class V, such as NCAM1, have also been described in rare cases of primary MN without evidence of systemic autoimmune disease [37]. More research is still required to understand the autoimmune comorbidities of MN and the precise differences in the clinical course and prognosis associated with the response against specific autoantigens. If confirmed, these findings are likely to lead to a new diagnostic paradigm in the evaluation of MN that is likely to involve a full serological panel testing against multiple known antigens (Table 3). 

The consensus is that patients with nephrotic-range primary PLA2R-positive MN generally require immunosuppressive treatment. In recent years three trials: GEMRITUX, MENTOR and STARMEN have shaped our approach to different IS regimens. GEMRITUX trial tested the non-immunosuppressive anti-proteinuric treatment (NIAT) against rituximab (RTX). As drugs targeting B-cells require longer time to achieve results, after initial disappointment, 17-month follow-up showed that rituximab was indeed effective in reducing proteinuria [38]. In MENTOR trial, RTX was superior to cyclosporine in inducing and maintaining complete or partial remission at 24 months (60% vs. 20%) [39]. Rituximab has even been proposed as first-line therapy [40], although some studies suggest that it may be less effective than the Ponticelli regimen in patients with high PLA2R values [37,41]. The STARMEN trial was designed to settle this issue: it compares a regiment of alternating corticosteroids + CYF to sequential tacrolimus + RTX treatment. Contrary to the initial hypothesis, steroid-CYF regimen yielded better efficacy, though with significantly higher rate of all adverse effects [42]. Other B-cell targeted drugs include ofatumumab, tested by Ruggenenti and Remuzzi in 11 patients with rituximab-resistant pMN [43] and a monoclonal antibody, belimumab, that in a clinical trial significantly reduced the PLA2R antibody levels during the 12 weeks of follow up [44]. 

An important topic in MN treatment is anticoagulation, as the nephrotic syndrome and the disease itself contribute to an increased risk of venous and arterial thrombotic events. Most guidelines are based on experts’ opinions, as data on this topic is scarce. The first step is an assessment of an individual risk using an online calculator available at med.unc.edu/gntools (accessed on 12 March 2021) (for venous thrombosis) and Framingham risk score combined with patients’ history and clinical data (for arterial emboli). As the risk of thrombotic events is related to the level of serum albumin, anticoagulation or/and aspirin should be considered in high-risk patients (albumin < 2.0–2.5 g/L). As no randomized controlled trials on this topic have been published, warfarin is still the drug of choice [45]. The use of heparin in the setting of potential antithrombin deficiency remains unclear.

## 5. Future Directions

There are two critical questions that a clinician has to answer when facing a patient with a biopsy diagnosis of membranous nephropathy. If the patient is PLA2R positive, then what is the risk of progression to kidney failure? If the patient is PLA2R negative, then is the disease is primary or secondary?

Some of these questions may be addressed by genetic studies. In a genome wide association study (GWAS) for primary MN (pMN) conducted in European patients, surprisingly strong genetic interactions were found for the HLA and PLA2R1 loci [46]. Researchers speculated that the PLA2R risk locus may increase the immunogenicity of the molecule, while a permissive (Human Leucocyte Antigen) HLA haplotype was associated with the production of autoantibodies [47]. A recent international genetic study of over 3700 patients demonstrated that four distinct genomic loci and their genetic interactions accounted for nearly one-third of the disease risk [48].

When combined with serum anti-PLA2R-Ab testing, the genetic risk score based on that study improved the discrimination of primary MN from controls and other forms of glomerulopathies. This approach significantly improved the sensitivity of serologic tests while maintaining high diagnostic specificity. The combined genetic and serologic testing is especially useful in the diagnosis of patients presenting with indeterminate PLA2R-Ab levels in the range of 2–20 units/mL, offering the promise to obviate the need for a kidney biopsy in this patient group.

A study by Seitz-Polski et al. points to one more way of risk assessment in MN patients. Epitope spreading is a process of broadening an immunological response from initial dominant epitope to secondary epitopes. In MN, the dominant epitope in most cases the N-terminal cysteine-rich domain (CysR). Through spreading, epitopes CTLD1 and CTLD7 lectin domains become involved. It has been hypothesized, that patients without spreading (antibody activity restricted to CysR) have lower proteinuria and a higher rate of spontaneous remission than those with CTLD reactivity [49]. However, evidence on this topic is lacking and needs to be further validated.

In parallel with the progress in serologic and genetic diagnostics, urinary biomarkers offer another potential risk stratification method. Recent data demonstrated that the urinary presence of PLA2R-Ab can be used to diagnose MN as successfully as serum-based methods. In a small study by Wang et al., 19 patients with pMN had a positive urinary PLA2R-Ab measurement, whereas serum PLA2R-Ab was found in 18 patients with pMN in an indirect immunofluorescence test, providing promising pilot data for this approach [50].

**Figure 4:**
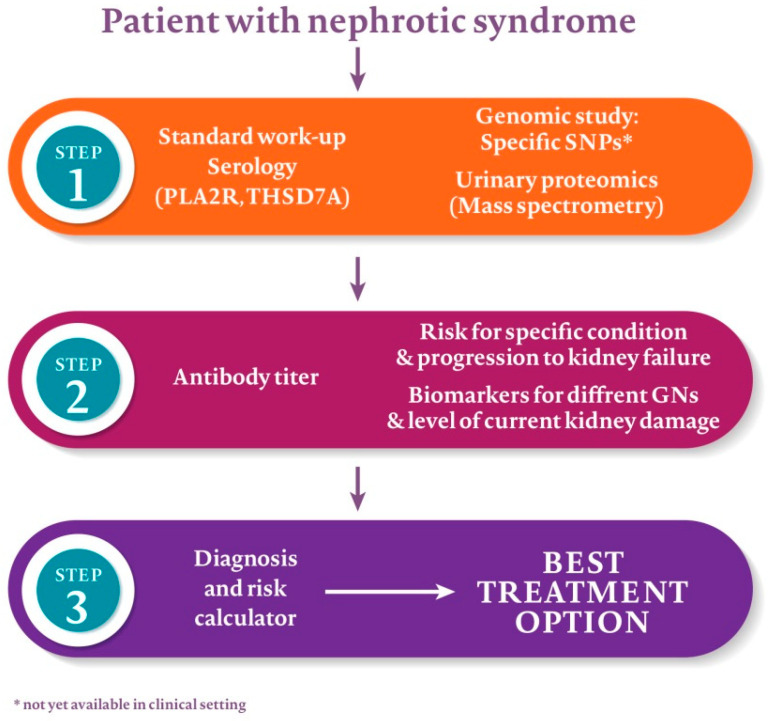
Possible future approach to a patient with nephrotic syndrome.

As serology became the milestone of MN research until recently the proteomic studies have been neglected. In kidney biopsy, deposits consisting primarily of IgG4 with C3 and C4 are more characteristic of pMN. Mixed IgG1-3 deposits with C1q component are found in other MN types. Studies using mass spectrometry (MS) provide a more detailed picture including all complement factors and pathways. For example, PLA2R- and exostosin-associated MN have similar spectral counts of C3-C9 proteins and different counts of complement regulating proteins: CFH, CFHR-1, -5 and vitronectin [51]. MS-based urinary proteomic analysis confirms complement activation and overexcretion of alpha-1-antitripsin and afamin in pMN [52]. The significance of such findings is yet to be determined. A combination of clinical data and biochemical markers (serum PLA2R level, urinary IgG, and low-/high-molecular weight proteins) might improve our risk stratification in MN patients (Figure 4).

## 6. Conclusions

Over the last decade, there have been several major advances in our understanding of the pathophysiology of MN. As a direct clinical consequence of these research developments, a significant group of patients with nephrotic syndrome may no longer need a kidney biopsy to establish the diagnosis of primary MN. The stressful observation period with countless 24-h urine collections and great uncertainty regarding the clinical course might soon be replaced by more precise risk stratification aided by disease activity monitoring with serial serum PLA2R measurements and more accurate prediction of the disease progression using genomics and urinary proteomics. We hope that this is also the future for other types of primary glomerular disorders.

## Figures and Tables

**Table 1:** Historical auto- and alloantigens in MN.

Antigen	Auto-/Alloantigen	Pathophysiology	Reference
Historical—‘proof of concept’ studies			
Megalin (rats only)	Autoantigen	Receptor in the tubular brush border in rodent kidneys, also expressed in rat podocytes	[1]
Cationic Bovine Serum Albumin	Alloantigen	Alloantigen (milk protein) that can plant in the GBM	[2,3]
Neutral endopeptidase	Alloantigen	NEP-deficient mothers produce IgG against fetal NEP; IgG migrates through placenta causing MN in infants	[5]

**Table 2:** Auto- and alloantigens in MN.

Antigen	Auto-/Alloantigen	Pathophysiology	Reference
PLA_2_R	Autoantigen	IgG_4_ (PLA_2_R-antibodies)-mediated reaction to phospholipase-2 receptor on podocytes	[6]
THSD7A	Autoantigen	IgG_4_ (THSD7A-antibodies)-mediated reaction to transmembrane glycoprotein on podocytes	[8]

**Table 3:** Recently reported antigens in MN requiring further validation.

Antigen	Available Data	Reference
NELL-1	Glycoprotein with a secretory sequence, not expressed by podocytes Serum antibodies identified under non-reducing conditions Predominantly IgG1 subepithelial and mesangial deposits Malignancy associated?	[33]
Protocadherin-7	Transmembrane protein No serum antibodies identified so far Predominantly IgG1 and IgG4 subepithelial deposits Elderly population?	[34]
Exostosin-1/-2	Transmembrane protein in endoplasmic reticulum No serum antibodies identified so far Predominantly IgG1 subepithelial and mesangial deposits Mainly lupus (class V) associated?	[36]
Semaphorin 3B	Secreted and membrane-bound protein Antibodies identified under reducing conditions only IgG 1–4 in predominantly subepithelial deposits Mainly pediatric cases?	[35]
NCAM-1	Glycoprotein of immunoglobulin superfamily Serum antibodies identified under non-reducing conditions IgG (all subclasses) subepithelial and mesangial deposits Mainly lupus (class V) associated?	[37]
